# A novel algorithm identifies stress-induced alterations in mitochondrial connectivity and inner membrane structure from confocal images

**DOI:** 10.1371/journal.pcbi.1005612

**Published:** 2017-06-22

**Authors:** Mathieu Ouellet, Gérald Guillebaud, Valerie Gervais, David Lupien St-Pierre, Marc Germain

**Affiliations:** 1 Département de Chimie, Biochimie et Physique, Université du Québec à Trois-Rivières, Trois-Rivières, Quebec, Canada; 2 Groupe de Recherche en Signalisation Cellulaire, Université du Québec à Trois-Rivières, Trois-Rivières, Quebec, Canada; 3 Centre de Recherche Biomed, Université du Québec à Trois-Rivières, Trois-Rivières, Quebec, Canada; 4 Département de Biologie Médicale, Université du Québec à Trois-Rivières, Trois-Rivières, Quebec, Canada; 5 Département de Génie Industriel, Université du Québec à Trois-Rivières, Trois-Rivières, Quebec, Canada; National Institutes of Health, UNITED STATES

## Abstract

Mitochondria exist as a highly interconnected network that is exquisitely sensitive to variations in nutrient availability, as well as a large array of cellular stresses. Changes in length and connectivity of this network, as well as alterations in the mitochondrial inner membrane (cristae), regulate cell fate by controlling metabolism, proliferation, differentiation, and cell death. Given the key roles of mitochondrial dynamics, the process by which mitochondria constantly fuse and fragment, the measure of mitochondrial length and connectivity provides crucial information on the health and activity of various cell populations. However, despite the importance of accurately measuring mitochondrial networks, the tools required to rapidly and accurately provide this information are lacking. Here, we developed a novel probabilistic approach to automatically measure mitochondrial length distribution and connectivity from confocal images. This method accurately identified mitochondrial changes caused by starvation or the inhibition of mitochondrial function. In addition, we successfully used the algorithm to measure changes in mitochondrial inner membrane/matrix occurring in response to Complex III inhibitors. As cristae rearrangements play a critical role in metabolic regulation and cell survival, this provides a rapid method to screen for proteins or compounds affecting this process. The algorithm will thus provide a robust tool to dissect the molecular mechanisms underlying the key roles of mitochondria in the regulation of cell fate.

## Author summary

Mitochondria are the main providers of cellular energy and as such, play a crucial role in many cellular processes whose deregulation are linked to both neurodegenerative diseases and cancer. In order to perform their functions, mitochondria dynamically regulate their length, assemble into interconnected networks and modulate their internal structure. Measuring these mitochondrial dynamics can thus provide important information about cellular state. Although some automated methods can provide a partial assessment of mitochondrial networks, the gold standard remains manual quantification, a time-consuming process. Here, we developed a new algorithm that accurately identifies both mitochondrial elongation and fragmentation occurring in response to different cellular stresses, as well as concomitant changes in mitochondrial connectivity and their internal structure. Given its sensitivity and ease of use, the algorithm will provide a robust tool to dissect the mechanisms by which mitochondria regulate cell fate.

This is a *PLOS Computational Biology* Methods paper.

## Introduction

Mitochondria play a crucial role in metabolic regulation and cell survival through the production of cellular energy, buffering of cytosolic calcium and regulation of apoptotic cell death [1,2,3,4,5]. These events are controlled by changes in mitochondrial dynamics, the continuous fusion and fission of the mitochondrial network [1,3]. For example, mitochondrial fragmentation and associated changes in mitochondrial innermembrane structure (cristae) promote efficient cytochrome c release during apoptosis and rapid cell death [6,7]. On the other hand, cellular stress such as starvation, acidosis, and UV irradiation increase mitochondrial length and promote tighter cristae, which are required for cell survival under these conditions [8,9,10,11,12]. In fact, impaired mitochondrial dynamics has been linked to a range of diseases, including neurodegenerative diseases such as Charcot-Marie-Tooth type 2A, dominant optic atrophy, Alzheimer’s disease and Parkinson’s disease [3,4,13].

While changes in mitochondrial morphology can provide important information about metabolic state and cell survival, the proper quantification of mitochondrial networks remains challenging. Two main approaches have previously been used to quantify mitochondrial dynamics: binning cells according to the overall aspect of their mitochondrial network (i.e. fragmented vs elongated), and measuring the length of individual mitochondria within the network.

The first approach, which can also be extended to the quantification of a number of specific mitochondrial structures present in each cell (such as blobs and donuts) [14, 15, 16, 17, 18, 19], provides a quick overview of the state of mitochondria within a cell population. It is the method generally used when manually measuring changes in mitochondrial networks [8,11], and can be automated through machine learning to successfully identify mitochondrial fragmentation caused by diverse treatments [14,16,18,19]. On the other hand, several types of stress cause mitochondrial elongation, a process that has recently emerged as crucial to maintain cell survival [8,10,11]. While the original experiments describing stress-induced mitochondrial fusion used manual binning [8,10,11], automating this quantification would greatly simplify data analysis and thus provide much needed insights into the processes regulating these events. However, as shape-based quantification (the first approach) was originally developed to quantify mitochondrial fragmentation [20,21,22], its ability to identify mitochondrial elongation remains unclear. In fact, there is an important caveat with this approach when measuring mitochondrial elongation. Contrary to fission, where fragmented mitochondria are easily identified as isolated round shapes, mitochondrial fusion observed upon starvation and other stresses requires arbitrarily identifying what constitutes a “normal” mitochondrial network. Binning of mitochondrial networks as elongated, intermediate or fragmented is then done according to this arbitrary threshold [8,11]. In addition, mitochondria vary in shape and length within a single cell, further complicating quantification [16,18,19]. Directly measuring the length of individual mitochondria (the second approach) and their connectivity could thus provide a more accurate quantification of mitochondrial networks under conditions of increased length and connectivity. This approach has however been limited by the prohibitive amount of time required to manually calculate mitochondrial length and the fact that the connectivity of the network renders the identification of its actual topology difficult or impossible.

Because of this, we used an automated probabilistic approach to quantify mitochondrial length and connectivity from confocal images of cells in culture. In this approach, instead of arbitrarily assigning a single topology to the mitochondrial network, we determined the set of possible configurations from which we calculated the most probable distribution. Using this method, we could identify starvation-induced mitochondrial elongation as well as fragmentation caused by genetic or chemical inhibition of mitochondrial function. We also provide evidence that the ratio of mitochondrial components (mitochondrial ends, tubules and junctions), as well as the number of these components in each cluster of mitochondria, provide a very sensitive measure of network connectivity. Furthermore, as changes in cristae structure are a key aspect of mitochondrial dynamics, we extended the method to measure changes in inner membrane versus outer membrane to provide a new fluorescence-based approach to screen cellular pathways modulating cristae structure. Using this novel approach, we showed that outer membrane fission can be separated from changes in inner membrane structure following Complex III inhibition. Overall, the algorithm we describe here provides a robust tool to dissect the molecular mechanisms underlying the mitochondrial regulation of key aspects of cellular functions, including metabolism, differentiation and cell death.

## Results

**Fig 1:**
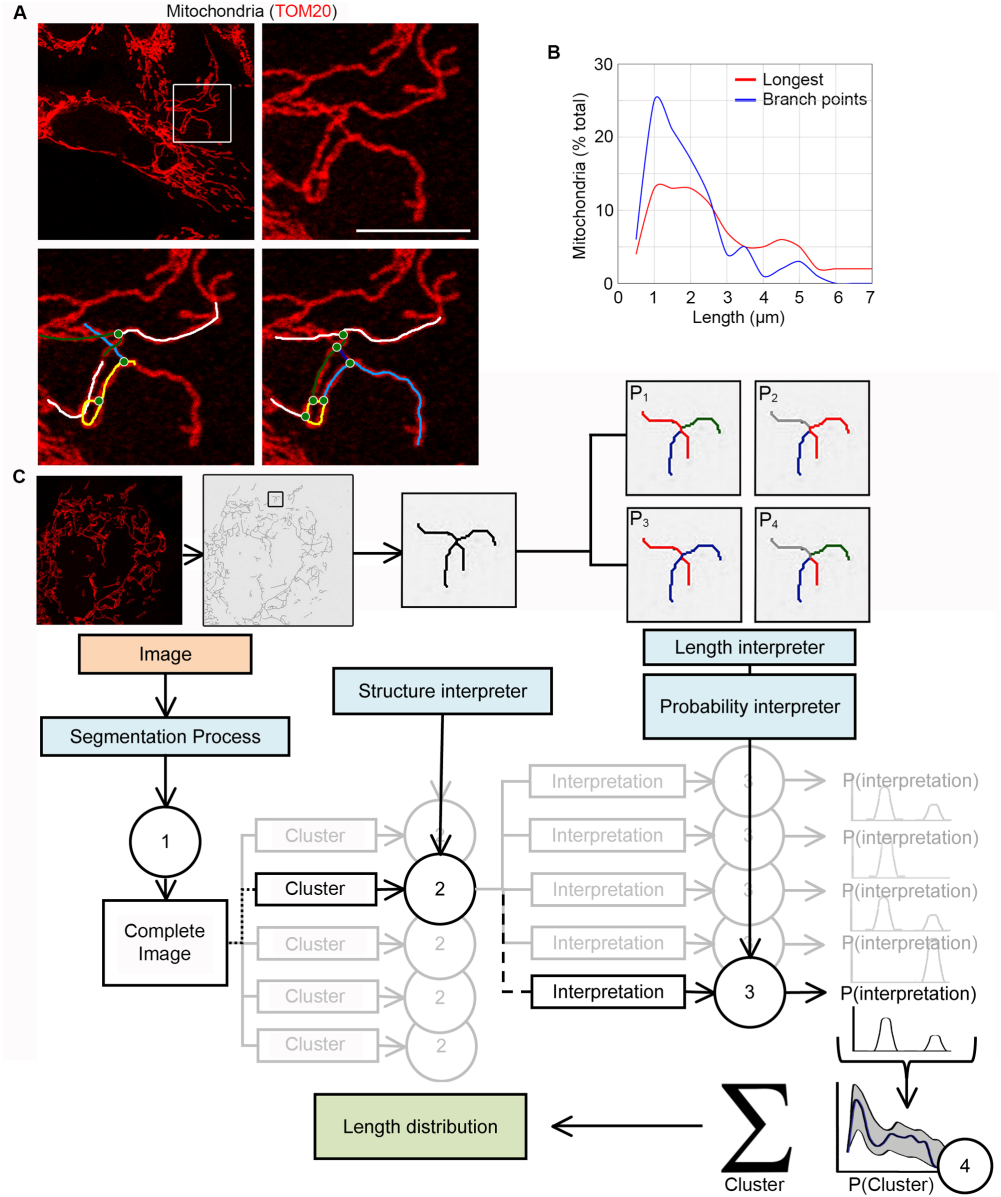
Overview of the algorithm. (A-B) Several distinct topologies can be assigned to a single mitochondrial network. U2OS cells were stained for the mitochondrial marker TOM20 and imaged. The lower panels show two distinct possible topologies for the same mitochondrial cluster (boxed area in top left panel (A)). Colour lines represent individual mitochondrial tubules; green circles, junctions (A). Mitochondrial length was then quantified from 7 cells by measuring the distance between each connexion (B; Blue) or the longest possible mitochondria to which the other mitochondria are connected (B; Red). (C) Overview of the analysis process. Grayscale confocal images are first converted into binary images (see Methods) then fed to the algorithm (1). The mitochondrial network is then separated into clusters of overlapping mitochondria that are analysed by the structure interpreter (2). The resulting structural information (ends, tubules, junctions) is then used to generate the probability of the different possible configurations for each cluster (3). The distributions are then used to generate the overall distribution probability for the cell (4). See also S3 Fig.

Changes in mitochondrial length and connectivity regulate key cellular processes including metabolism, cellular differentiation and apoptosis [1,2,3]. However, the tools required for the proper quantitative analysis of mitochondrial dynamics are limited, especially for elongated networks. These tools generally rely on crude measures such as the aspect ratio of individual mitochondria or the identification of branching points between mitochondria [21,22,23,24]. Thus, most quantification remains done manually [8,10,11]. This approach has however been severely limited by the difficulty to properly assess the topology of the mitochondrial network, especially since most images do not contain the information required to determine whether two overlapping mitochondria are connected or just cross each other. This is exemplified in Fig 1A where the mitochondrial network could be described in several ways that affect both the number of branching points (Fig 1A, green circles) and length of individual mitochondrial branches (Fig 1A, coloured lines). In fact, quantification of mitochondrial length by measuring the distance between branching points [23] leads to a very different length distribution than considering the longest possible mitochondria within the network to which to connect the other mitochondria (Fig 1B).

As the actual length distribution within the mitochondrial network likely falls somewhere between these two extremes, we developed a probabilistic approach to measure the distribution of mitochondrial length and connectivity. In this approach, the set of possible network configurations are determined and used to generate the most probable mitochondrial length distribution. The overview of the process is shown in Fig 1C. Confocal microscope images of mitochondria are first segmented (converted into binary images; see Methods) then fed to the algorithm, which generates a skeleton representing the mitochondrial network and identifies structural elements of this network (mitochondrial tubules, junctions, ends). The set of possible connection patterns linking these structural elements is then determined and compiled to generate the overall distribution of the mitochondrial network. To facilitate analysis, network configuration is determined by first separating mitochondria in smaller clusters that correspond to a set of mitochondria that are connected or overlapping, but are independent of the rest of the network (Fig 1C; S3 Fig). Once determined, the configuration of each cluster is summed to generate the distribution of the entire network. In addition, as this output represents the most probable configuration, the result is express within a confidence interval (Blue line, average; Confidence interval, grey; Fig 1C).

### Validation of the algorithm using synthetic images

To determine the accuracy of the algorithm, we first tested it against a set of synthetic, computer-generated images containing mitochondria with length distributions and densities within the range found in normal fibroblasts (1–10 μm in length; see for example [10]). For each synthetic image, we randomly generated a number of 1 pixel wide mitochondria with lengths within the specified range. The width of this skeleton was then increased to match that of actual mitochondria, a step required to generate images that were equivalent to those of real cells. These images were then fed to the algorithm, which generates a new skeleton as the first step of the analysis.

Because the density of mitochondria varies within each cell (S1A and S1B Fig), we first determined the effect of mitochondrial density on the accuracy of the skeleton generated by the algorithm. To manipulate mitochondrial density, we used images with the same size (500 x 500 pixels) but with an increasing number of mitochondria: 100 mitochondria (within the range of mitochondrial densities found in the periphery of cells) to 500 mitochondria (mitochondrial density equivalent to the denser perinuclear region) (S1A and S1B Fig). We then compared the original 1-pixel wide mitochondria to the algorithm-generated skeleton over a range of densities (S1C Fig; a ratio of 1 indicates same position and same length). Importantly, the ratio was close to 1 for the images within the range of mitochondrial densities found in cells, indicating that the skeletinization process function properly within this range. However, the ratio dropped abruptly when the area occupied by mitochondria was above 60% (S1C Fig). While we did not observe such a high mitochondrial density in actual cells, we nevertheless excluded any such cluster from the analysis to avoid spurious data. These non-analysed clusters represent a very small fraction of the total clusters in all cases we tested (<5%; S1D Fig).

**Fig 2:**
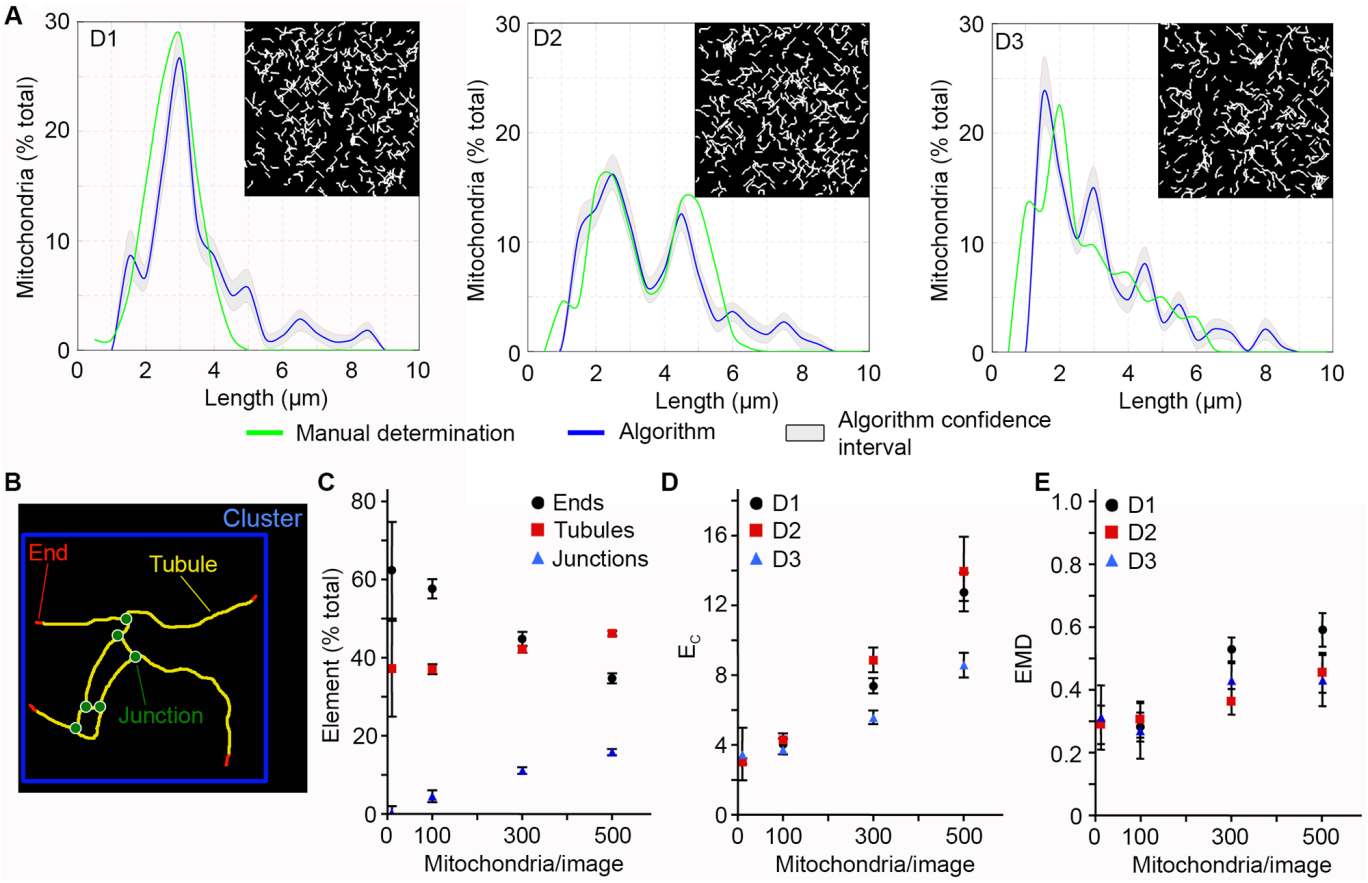
The algorithm identifies mitochondrial length and connectivity in computer-generated images. Three distinct types of distributions were used to test the algorithm: a simple Gaussian (D1), a double Gaussian (D2) and a distribution reflecting the distribution found in fibroblasts (D3). Each image (500 pixels x 500 pixels) contains a defined number of mitochondria (between 10 and 500). A representative example for each distribution (300 mitochondria) is shown in (A) with the input distribution in green, the calculated distribution in blue and the confidence interval in grey. (B) Schematic representation of the different structural element of the mitochondrial network that are analysed by the algorithm. (C-D) Mitochondrial connectivity increases with the number of mitochondria in computer-generated images. (C) The proportion of each type of element was calculated in D3 distributions with increasing number of mitochondria and expressed as a percent of the total number of structural elements ± S.D. of at least 45 images/condition. (D) Alternatively, we calculated *E*_*C*_ values for the 3 distribution types. (E) EMD values between the input distribution and the algorithm-generated distribution for the different distributions types. Shown is the average of at least 45 images/condition ± S.D.

Having validated the skeletonization process, we then tested the ability of the algorithm to analyse different mitochondrial distributions. Three different length distributions were tested: a simple Gaussian distribution with an average mitochondrial length of 3 μm (D1), a double Gaussian distribution centered at 2.5 μm and 5 μm (D2) and a distribution reflecting the length distribution found in normal fibroblasts (D3) (Fig 2A; Green lines). The D1 and D3 distributions were chosen as they represent distributions that are found in actual cells (D3, normal mitochondrial distribution; D1, fragmented mitochondria) [10, 25]. A representative example of each distribution containing 300 mitochondria is shown in Fig 2A, with the green line representing the actual distribution within the image and the blue line, the distribution calculated by the algorithm with the confidence interval in grey.

Mitochondrial networks are defined by both the length and connectivity of their mitochondria. We thus took advantage of the images with varying mitochondrial densities generated above to create mitochondrial networks with increasing network connectivity. We then used several parameters to measure network connectivity in each image. Three types of structural elements are present in each mitochondrial cluster: mitochondria ends, tubules and junctions (Fig 2B). The proportion of each element varies with the number of connection that mitochondria make: single mitochondria contain one tubule and two ends while the number of junction increases with connectivity. For example, in a D3 distribution containing only a small number of mitochondria (10), there are no junctions and the end to tubule ratio is such that most mitochondria are a single tubule with 2 ends (1 tubule/2 ends; Fig 2C). Increasing the number of mitochondria from there resulted in an increased number of junctions concomitant with a decrease in the number of ends (Fig 2C).

In addition to measuring the ratio of each type of element present in these images, it is possible to globally quantify connectivity by asking how many structural elements are present in each cluster, the number of element/cluster (*E*_*C*_) increasing with connectivity. The minimum value for *E*_*C*_ is found in isolated mitochondria. In our computer-generated images containing 10 mitochondria, each mitochondrion was isolated from the others, resulting in *E*_*C*_ values of 3 (1 tubule + 2 ends, Fig 2D). As with individual elements, *E*_*C*_ values increased with mitochondria numbers for all distributions (Fig 2D). Interestingly, while individual elements varied linearly with mitochondria number, *E*_*C*_ values increased more rapidly (Fig 2D), suggesting that *E*_*C*_ is a sensitive measure of overall network connectivity.

Having established the properties of our computer-generated images, we quantified the variation between the calculated and actual distributions for each image using the earthmover distance (EMD) as a measure of the distance between two distributions. EMD measures the amount of work required to transform one distribution into a second one, the work being defined as the area moved multiplied by the distance traveled [26,27,28]. A larger EMD score therefore reflects a greater discrepancy between two distributions. For example, the difference between the mitochondrial distributions considering branching points or the longest mitochondria (Fig 1B) corresponds to an EMD of 1.25. Actual cells contain a wide range of mitochondrial sizes, which varies according to cell type and metabolic conditions. Because these variations are linked to metabolic changes, cell death and cellular proliferation and differentiation [8,12], it is important to be able to accurately identify a range of mitochondrial distributions and connectivity. We therefore used EMD scores of computer-generated images to define how the density of mitochondria and the number of branching points affect the ability of the algorithm to determine proper mitochondria distribution. Because distributions with 10 mitochondria do not contain junctions (Fig 2C), there are no uncertainties in their distributions as determined by the algorithm. The EMD value of these images therefore represents the minimal EMD score possible for this experimental setting (background), a score that results from small changes in mitochondrial length caused by the segmentation process applied to the images before their analysis by the algorithm. From there, EMD scores initially increased with increased number of mitochondria for all distributions, but rapidly reached a plateau (Fig 2E) even though connectivity continued to increase (Fig 2C and 2D). This suggests that the accuracy of the algorithm is only minimally affected by changes in distribution pattern and connectivity and should therefore function across a range of network topologies.

### Empirical validation of the algorithm

**Fig 3:**
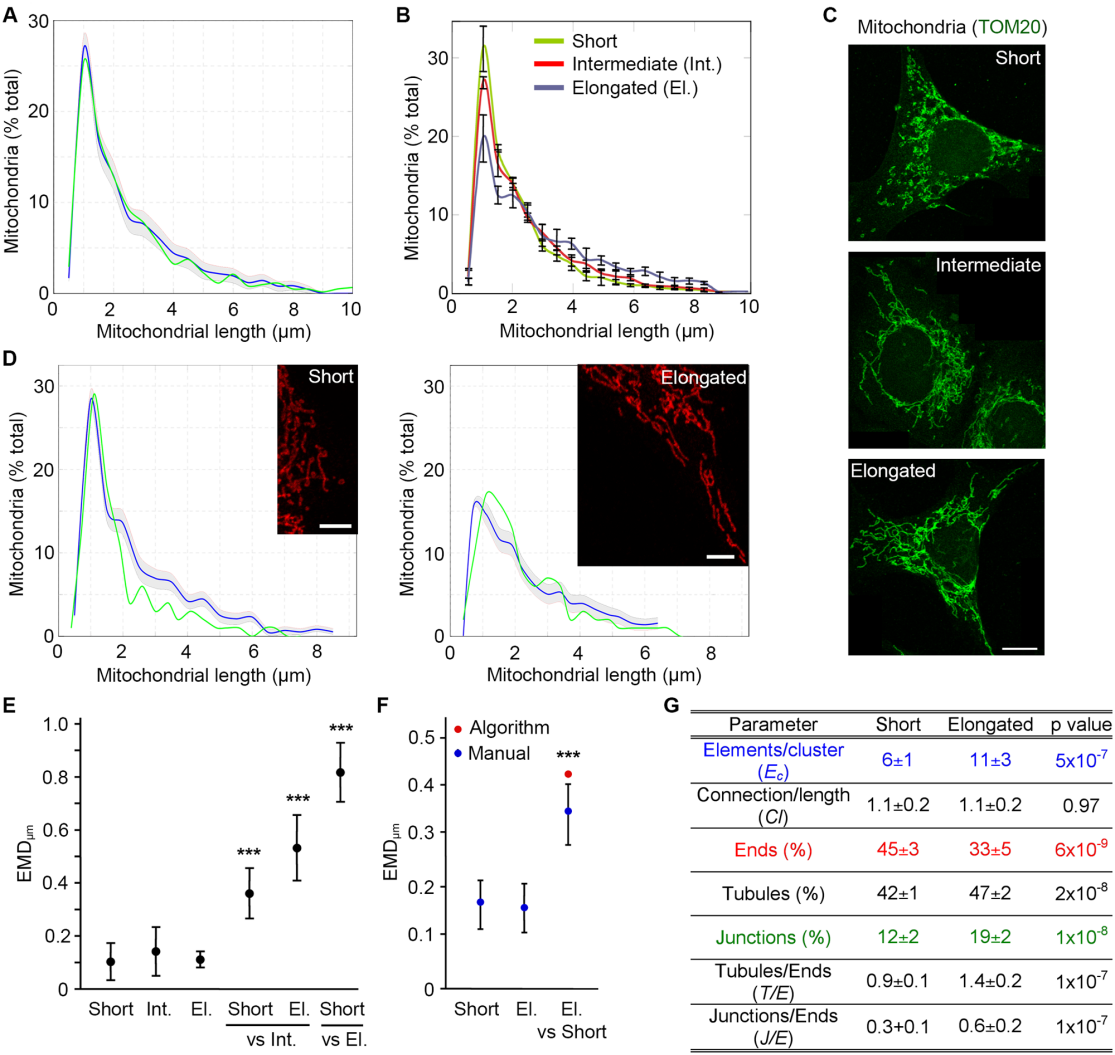
The algorithm identifies mitochondrial length and connectivity in sample images from cells. (A-D) MEFs were stained for the mitochondrial marker TOM20 and imaged. Mitochondrial length was then determined in 8 whole cells manually (Green line) and using the algorithm (Blue line, confidence interval in grey) (A). Cells were then binned as containing short, intermediate or elongated mitochondria and analysed with the algorithm (B, distribution (3 cells/type ± SD); C, representative images). Scale bars 10 μm. Alternatively, we manually quantified mitochondrial length in portions of cells with well-defined mitochondria and classified them as short or elongated mitochondrial networks. Images from the same category were pooled to generate a mitochondrial profile (D; Green, manual determination; Blue, algorithm; Grey, confidence interval). Manual counts represent the average of at least 5 images. (E-F) EMD quantification of the differences between distribution types in whole cells (E) and image sections (F). EMD values were first calculated between each cell within a distribution type and the average for that distribution, to calculate the experimental variation (first 3 data points (E); first 2 data points (F)). To calculate the EMD between distribution types, each cell within a distribution type was then compared to the average for each of the other distributions (Short/El. vs Nit and Short vs El. (E); El. vs Short (F)). Data is expressed as the average EMD value ± S.D. *** p<0.001. El.: Elongated; Int.: Intermediate. (G) Parameters for the short and elongated mitochondrial networks in (B-C).

As our results indicate that the algorithm accurately identifies mitochondrial lengths within a large range of synthetic images, we then validated it on actual mitochondria. To provide a baseline to which to compare the algorithm, Mouse Embryonic Fibroblasts (MEFs) were marked with the mitochondrial marker TOM20 and imaged. We first manually measured mitochondrial lengths within whole MEFs (n = 8) and compared the result to the algorithm output. As with the synthetic images, the blue line represents the distribution determined by the algorithm and the grey zone the confidence interval of the algorithm (1 SD) (Fig 3A). The distribution of mitochondrial length measured manually (Fig 3A; green line) almost completely overlapped with the one determined by the algorithm (EMD value of 0.13 between the manual count and the algorithm)

Independently of any treatment, mitochondrial networks can range from fragmented to highly elongated and connected (tubulated), although the latter is more prominent during starvation [8,11]. To determine the ability of the algorithm to accurately identify the full range of mitochondrial length distributions, we analysed mitochondrial length in individual cells using the algorithm. For this, we generated a set of images from control and starved MEFs from which we identified cells with the longest mitochondria (elongated) as well as cells with short mitochondria and cells with an intermediate (“normal”) phenotype (Fig 3B and 3C). Consistent with the manual binning of the images, the algorithm identified a distinct length patterns for each type of mitochondrial network (Fig 3B; error bars represent the variability between individual cells (SD, n = 3)). We also generated a set of smaller images corresponding to sections of cells with well-defined mitochondria for which the lengths were measured manually. These images were then binned into either short or elongated networks (compare Fig 3B with Fig 3D) and length profiles determined by the algorithm. However, as each of these smaller images contained a limited number of structural elements, we concatenated the individual images within a distribution to give statistical weight to each sample and thus improve analysis accuracy. The algorithm output was then plotted for each distribution type, with the blue line representing the calculated distribution and the grey zone the confidence interval (1 SD) of the algorithm (Fig 3D). As with the whole cells, the mitochondrial distributions generated by the algorithm matched the manual counts (Fig 3D).

The visual representation of mitochondrial distributions (Fig 3A, 3B and 3D) provides a rapid assessment of changes in mitochondrial length but does not directly allow quantifying these changes. We thus used EMD scores to quantify the differences between the distributions. Of note, as these scores were calculated based on the length in μm rather than the pixels used in Fig 2, their values cannot be directly compared to those of Fig 2 and are thus labelled EMD_μm_. As EMD are relative, we first calculated the experimental variation within each distribution as a baseline to compare the changes occurring between distributions. To that end, we calculated EMD_μm_ scores for each image within a distribution compared to the average for that distribution. This experimental variation was below 0.2 for all mitochondrial distributions examined (Fig 3E, first 3 data points; Fig 3F, first 2 data points). In contrast, EMD scores were significantly higher when comparing distinct distributions, both in whole cells (Fig 3E, Short or Elongated (El.) vs Intermediate (Int.); Short vs Elongated (El.)) and the smaller image sections (Fig 3F, Elongated (El.) vs Short). Furthermore, the EMD score for the algorithm-generated length distributions of cell sections was similar to the one between the manually quantified image sections (Fig 3F). EMD score variability (SD) could however not be calculated for the image sections analysed by the algorithm, as these were concatenated before analysis. Altogether, these results indicate that the algorithm accurately identifies changes in mitochondrial length. Importantly, this change can be quantified by calculating the EMD score between two distinct distributions, providing a simple measure of the global differences between distributions.

In addition to mitochondrial length, changes in the connectivity of the mitochondrial network are associated with cellular responses to stress [8,12]. This aspect of mitochondrial dynamics is however less well understood as existing tools rarely quantify connectivity. We thus used the parameters described above to quantify the differences in connectivity between the short and elongated distributions in Fig 3B. Consistent with increased connectivity, the number of tubules and junctions were increased in cells with elongated mitochondria, concomitant with a decrease in mitochondria ends (Fig 3G). We also measured the ratio of junctions/ends (*J/E*) and tubules/ends, which were more sensitive than the individual measures (*J/E* was doubled; Fig 3G). In addition to these changes in specific structural elements, global connectivity was also increased as measured by an increase in *E*_*C*_ (Fig 3G). In contrast, the number of connections/length did not correlate with these changes (Fig 3G), suggesting that it is not a sensitive measure of connectivity. Overall, our results indicate that the algorithm accurately identifies distinct mitochondrial lengths and connectivity in both computer-generated images and images from actual cells.

### Starvation increases mitochondrial connectivity

**Fig 4:**
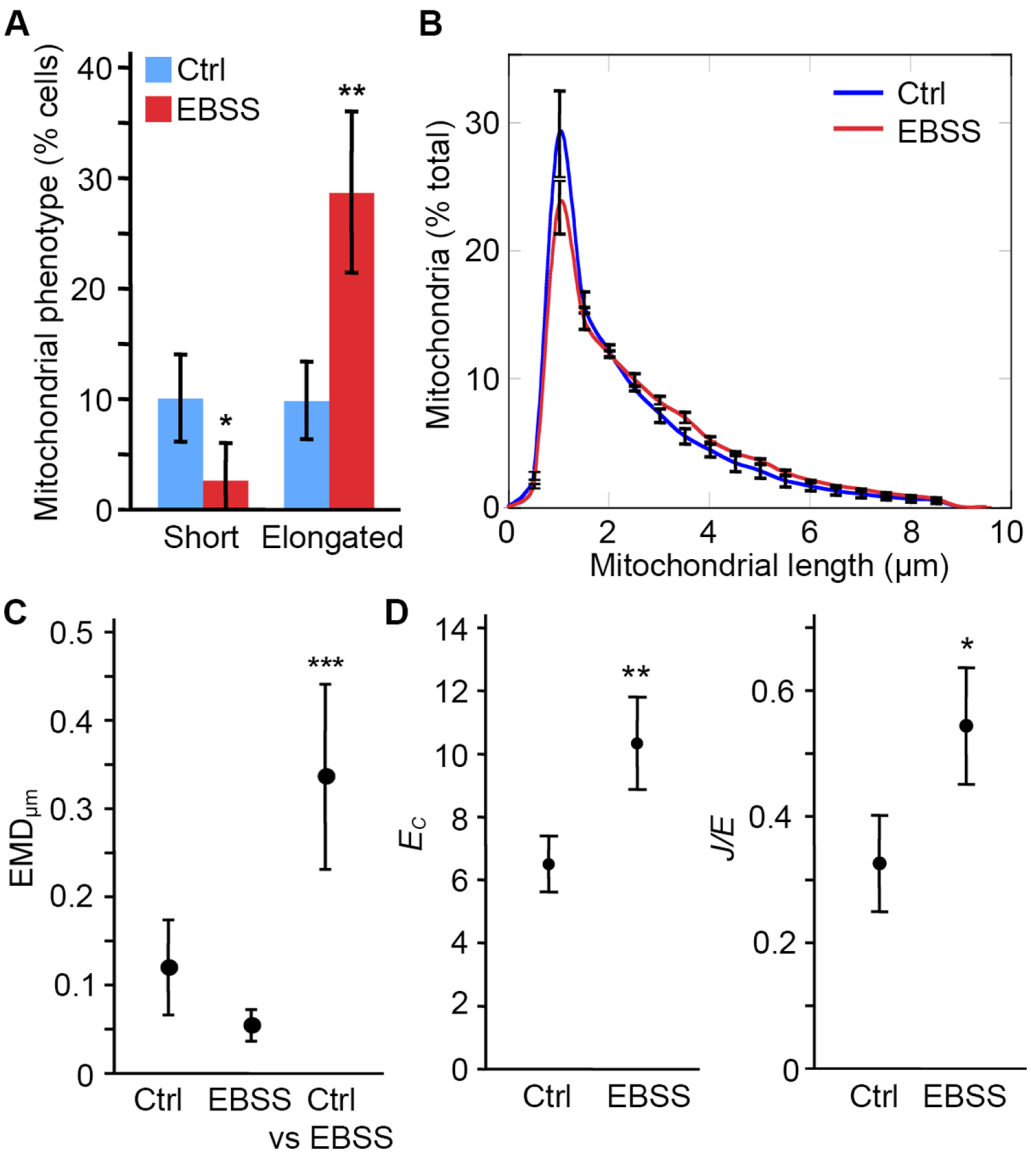
Starvation increases mitochondrial length and connectivity. To induce a starvation response, MEFs were incubated for 4 hours in EBSS. Control and EBSS-treated cells were then stained for the mitochondrial marker TOM20 and imaged. (A) Individual cells were manually counted as having a short, intermediate or elongated mitochondrial network. Data is expressed as the average % of cells within each category ± S.D.; n = 3 independent experiments. (B) Quantification of mitochondrial length by the algorithm. Data is shown as the average of 3 independent experiments ± SD. (C) EMD quantification of the distribution shift between control and EBSS-treated cells. The experimental variation was first calculated by comparing the average distribution of each experiment (n = 3) to the average of the 3 experiments for a same treatment (Ctrl, EBSS). The variation between Ctrl and EBSS was then measured by comparing the distribution of a given treatment in individual experiments to the average distribution for the other treatment (Ctrl vs EBSS). Data is expressed as the average EMD value ± S.D. *** p<0.001 (D) Starvation increases both *E*_*C*_ and *J/E*. Data is expressed as the average of 3 experiments ± S.D. * p<0.05; ** p<0.01.

Mitochondrial length and connectivity are regulated by metabolic state. For example, in response to amino acid starvation, mitochondria elongate to improve their bioenergetics output and prevent them from being degraded by mitophagy [8,10,11]. To determine whether the algorithm can quantify this response, we measured changes in the mitochondrial network triggered by decreased nutrient availability. Incubation of cells in EBSS (amino acid-free media) results in mitochondrial elongation in several cell types [8,10,11]. In our hands, a four hour incubation of MEFs in EBSS causes a mild but significant elongation of their mitochondria [10]. This can manually be measured by quantifying the number of cells with short mitochondria versus elongated mitochondria, with EBSS treatment decreasing the number of cells with short mitochondria and increasing cells with elongated mitochondria (Fig 4A). Consistent with this, we found a significant elongation of the mitochondrial network in EBSS-treated cells, which is observed as a shift in the algorithm-generated distributions shown in Fig 4B. Of note, as these experiments addressed the cellular response to metabolic changes rather than the accuracy of the algorithm, the error bars refer to the variability between experiments (SD, n = 3; at least 20 cells/condition in each experiment) and not the confidence interval of the algorithm.

The increase in mitochondrial length triggered by starvation prevents mitophagy and allows the distribution of fatty acids destined to ß-oxidation within the mitochondrial network [8,29]. Quantifying the global changes in the network is thus more informative that assessing the differences at specific lengths as previously done for mitochondrial fragmentation [25]. We therefore used EMD scores to quantify the global changes in mitochondrial distribution observed following EBSS treatment. Consistent with the test images (Fig 3E and 3F), each growth condition had an EMD_μm_<0.15 when comparing each experiment to the average value for the same condition (Fig 4C; Ctrl, EBSS) indicating low experimental variability. Importantly, EMD_μm_ was much larger when comparing EBSS mitochondria to control mitochondria (Fig 4C, Ctrl vs EBSS), consistent with EBSS causing mitochondrial elongation.

Mitochondrial elongation has been suggested to be associated with increased network connectivity following some forms of cellular stress, although this was not quantified [12]. To quantify these changes following EBSS treatment, we measured *E*_*C*_ and *J/E*. Concomitant with mitochondrial elongation, EBSS treatment increased both parameters (Fig 4D). This indicates that EBSS treatment promotes mitochondrial connectivity in addition to mitochondrial elongation.

### Selective effect of mitochondrial inhibitors on mitochondrial fragmentation

**Fig 5:**
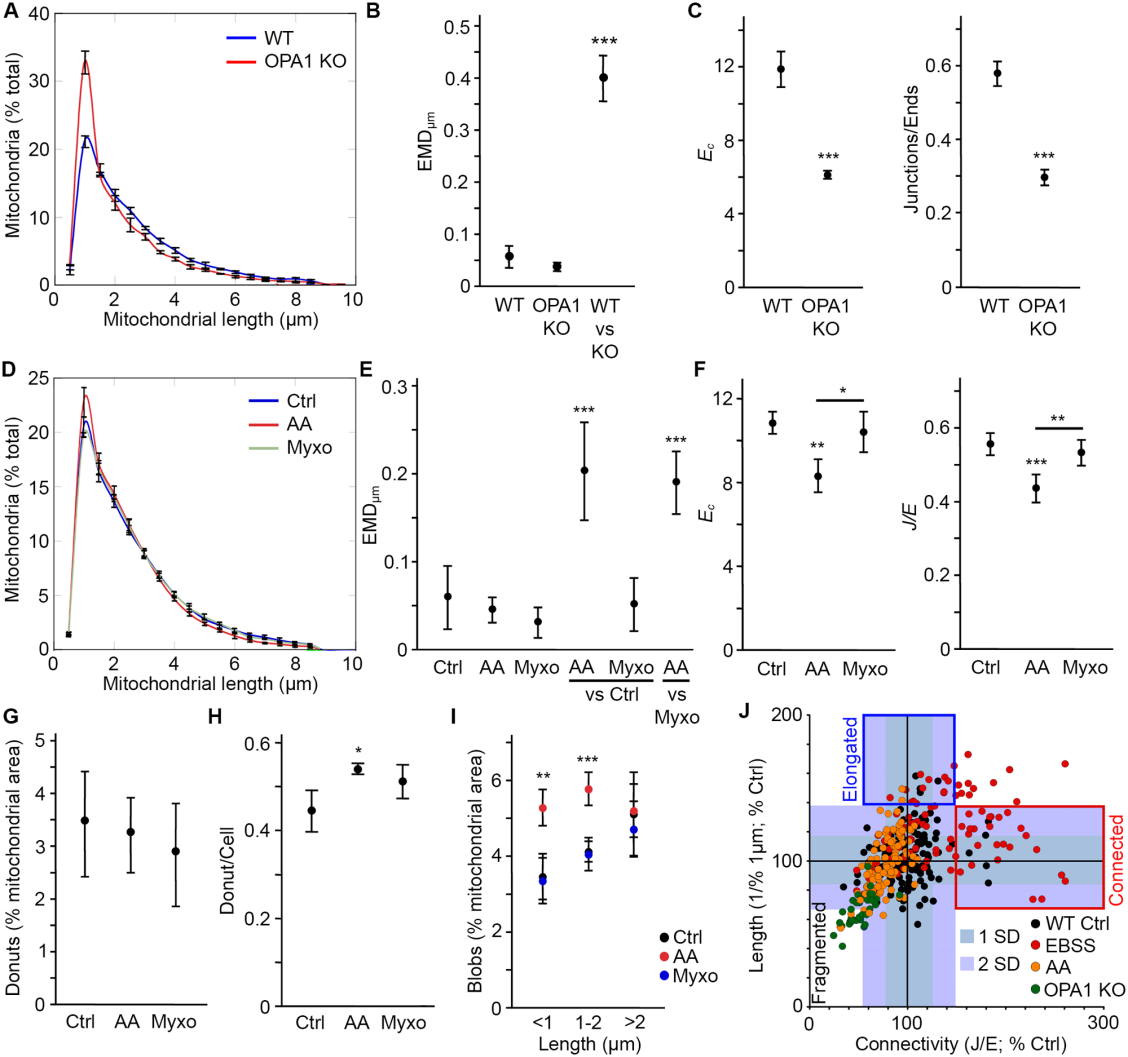
AA but not Myxo induces mitochondrial fragmentation in U2OS cells. (A-B) OPA1 KO mitochondria are fragmented. WT and OPA1 KO MEFs were stained using the mitochondrial marker TOM20 and imaged. Mitochondrial distributions (A) and EMD quantification of the shift in distribution between WT and KO cells (B; WT and OPA1 KO, experimental variation; WT vs KO, variation between genotypes) were then determined as for EBSS treatments (Fig 4). Data is expressed as the average of 3 independent experiments ± SD. *** p<0.001. (C) Changes in *E*_*C*_ and *J/E* correlate with mitochondrial fragmentation. Data is expressed as the average of 3 independent experiments ± S.D. *** p<0.001. (D-E) Decreased mitochondrial length in U2OS cells treated with the Complex III inhibitor AA. Cells were treated for 4 hours with AA or Myxo, then fixed and analysed as in (A-B). Data is expressed as the average of 3 independent experiments ± SD. *** p<0.001. For EMD scores (E), Ctrl, AA and Myxo refer to the experimental variation while AA/Myxo vs Ctrl and AA vs Myxo measure the change in distribution between treatments. (F) AA, but not Myxo, decreases mitochondrial connectivity as measured by *E*_*C*_ and *J/E* values. Data is expressed as the average of 3 independent experiments ± S.D. *** p<0.001, ** p<0.01, * p<0.05 compared with the control or AA where indicated. (G-I) Changes in specific mitochondrial structures following AA treatment. Donuts (mitochondria looped on themselves) were measured by the algorithm (G) or manually (H) and the result expressed as the average of 3 independent experiments ± S.D. Blobs (isolated mitochondria (*E*_*C*_ = 3) with a length <1 μm) were measured using the algorithm (I). Short isolated mitochondria with lengths between 1 and 2 μm, were also increased following AA treatment. Data is expressed as the average of 3 independent experiments ± S.D. *** p<0.001, ** p<0.01 compared to control. (J) Relationship between connectivity and length across different experimental conditions in individual cells. *J/E* ratios were used as the connectivity parameter. For length, we used the number of mitochondria in the 1 μm bin because this value correlates well with mitochondrial fragmentation. However to have an increasing value with increasing length, we used 1/this number. To allow comparison between experiments, all values were normalised to the control for that experiment. The shaded areas represent 1 SD and 2 SD from the control values. Individual cells from at least 3 experiments/conditions are shown.

The above results indicate that the algorithm can accurately identify an increase in mitochondrial length and connectivity as it occurs following nutrient depletion. We then determined whether it also identifies mitochondrial fragmentation caused by disruption of mitochondrial function. For this, we used two distinct models of loss of mitochondrial function: chemical inhibition of Complex III and genetic deletion of the essential mitochondrial protein OPA1 [8,10,30,31]. As OPA1 is required for mitochondria inner membrane fusion and maintenance of cristae structure [8,10,31], its deletion causes the fragmentation of the mitochondrial network. This was detected by the algorithm as an increase in short mitochondria (Fig 5A). This shift was significant as determined by the large EMD score between WT and OPA1 KO MEFs (Fig 5B, WT vs KO). In addition, both connectivity parameters (*E*_*C*_ and *J/E*) were greatly decreased in OPA1 KO MEFs (Fig 5C), reflecting the fragmentation of the mitochondrial network.

In addition to the deletion of essential mitochondrial proteins, inhibition of mitochondrial activity can be achieved using chemical inhibitors of the electron transport chain. Here, we used the Complex III inhibitor Antimycin A (AA) to inhibit mitochondrial function in U2OS cells, leading to reduced mitochondrial length (Fig 5D). As with the OPA1 KO MEFs, AA treatment caused a significant shift in mitochondrial distribution as determined by the large EMD score between control and AA-treated cells (Fig 5E, AA vs Ctrl). We also used a second Complex III inhibitor, Myxothiazol (Myxo), which inhibits electron transfer at the Q_o_ site rather than the Q_i_ site inhibited by AA [32]. Interestingly, while Myxo induces the production of mitochondria-derived vesicles and large lysosomal vacuoles [33], two signs of mitochondrial dysfunction, it did not cause mitochondrial fragmentation (Fig 5D and 5E (Myxo vs Ctrl for EMD score)). This suggests that simply inhibiting mitochondrial function is not sufficient to cause mitochondrial fragmentation. To substantiate these findings, we then measured the connectivity parameters for both inhibitors. Consistent with the length data, only AA caused an important decrease in *E*_*C*_ (Fig 5F). Similarly, the *J/E* ratio was decreased following AA treatment but not exposure to Myxo (Fig 5F).

Previous studies [14,15,17,18] used the occurrence of specific mitochondrial structures within cells as a measure of mitochondrial dysfunction in response to stress. As these structures are directly related to the structural elements the algorithm measures, we then extracted this data. The two main reported structures are donuts (a mitochondrion looped on itself to form a circular structure [14,15,17,18]) and blobs (completely fragmented mitochondria [14,18]). We first measured donuts, which we defined as a junction connected twice to the same tubule, the third connection leading to a mitochondrial end. Using this criterion, we found a very small number of donuts in U2OS cells irrespective of the experimental condition (Fig 5G), a finding that was confirmed by manual counting (1 donut/2 cells; Fig 5H). In contrast, the area covered by Blobs (defined as a cluster with 3 structural elements (1 tubule, 2 ends; *E*_*c*_ = 3) and a length less than 1 μm) was significantly increased in AA-treated cells (Fig 5I). As this increase in Blobs did not occur in Myxo-treated cells (Fig 5I), this further supports the idea that Myxo does not cause significant mitochondrial fragmentation. Interestingly, clusters with an *E*_*c*_ = 3 but with length up to 2 μm were also increased in AA-treated cells (Fig 5I), suggesting that the loss of connectivity measured by *E*_*C*_ and *J/E* extends beyond mitochondrial fragmentation. This and the fact that AA does not cause complete fragmentation of the mitochondrial network prompted us to analyse the relationship between mitochondrial length and connectivity.

To determine whether length and connectivity are correlated, we plotted their normalised values for individual cells (Fig 5J). For each parameter, we also indicated the average and SD for control cells to highlight the effect of the treatments (Fig 5J). 25% of EBSS-treated cells showed increased connectivity without a concomitant increase in length (Fig 5J; Connected) while 13% had increased mitochondrial length but no change in connectivity (Fig 5J; Elongated). Similarly, while some AA-treated cells clustered with the OPA1 KO mitochondria, a portion of them showed decreased connectivity but normal length (Fig 5J), consistent with the presence of unconnected mitochondria with lengths up to 2 μm (Fig 5I). While these results are consistent with the average values presented above, they indicate that mitochondrial length is not directly correlated with connectivity. This suggests that length and connectivity are two independent determinants of mitochondrial networks.

### Identification of inner mitochondrial changes using the algorithm

Changes in mitochondrial length are associated with cristae rearrangements that play a crucial role in the regulation of metabolism and cell survival. For example, apoptosis-induced fragmentation leads to cristae widening and efficient cytochrome c release, while starvation-associated elongation promotes tighter cristae and improves mitochondrial efficiency [6,7,8,10]. While these changes are usually quantified by electron microscopy, it is possible to observe them by immunofluorescence staining of outer membrane and inner membrane markers under some circumstances [34,35,36,37]. Examples of this include mitochondrial swelling in AIF KO mitochondria and the inhibition of mitochondrial outer membrane fission in DRP1 mutant worms [35,37]. We thus determined whether the algorithm could detect the previously reported changes in mitochondrial matrix occurring in AA-treated mitochondria [38].

**Fig 6:**
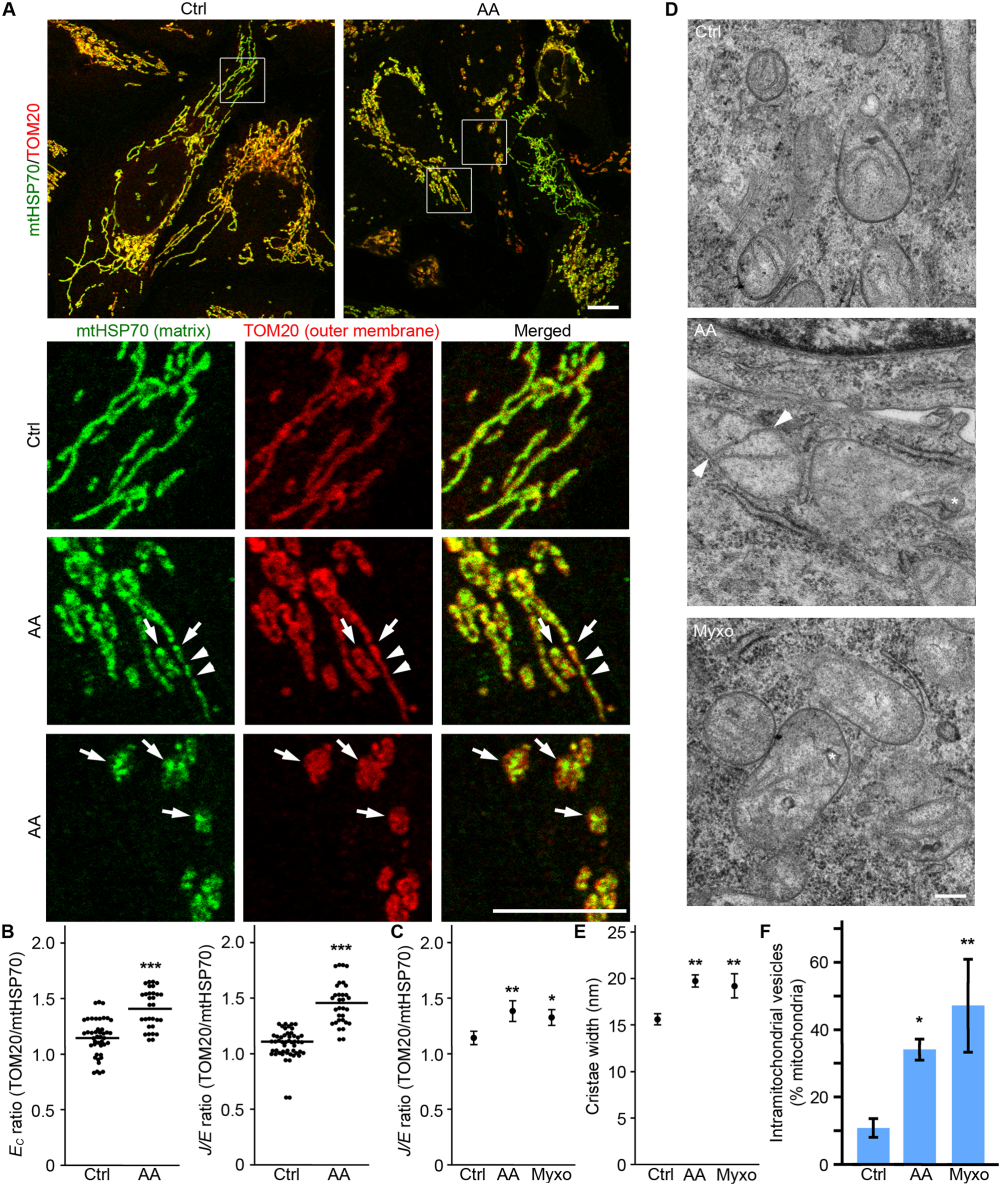
Differences in outer and inner membranes can be used to detect changes in cristae/matrix structure. U2OS cells were treated for 4 hours with AA or Myxo, then fixed and stained for the outer membrane marker TOM20 (Red) and the matrix protein mtHSP70 (Green). Representative images are shown in (A). Arrows denote sites where mtHSP70 accumulates; arrowheads, sites of inner membrane fission where TOM20 staining remains continuous. Scale bar 10 μm. (B) Quantification of the difference in *E*_*C*_ and *J/E* ratios between TOM20 and mtHSP70 staining. These parameters were calculated from control mitochondria with overlapping signals and AA treated cells showing distinct patterns of TOM20 vs mtHSP70 staining (as in the lower two panels in (A)). Data is expressed as the ratio of *Parameter(TOM20)*/*Parameter(mtHSP70)*. (C) *J/E* ratio is increased following Complex III inhibition. Data is expressed as the average of 4 independent experiments ± S.D. ** p<0.01, * p<0.05. (D-F) Mitochondrial untrastructure changes are present following Complex III inhibition. U2OS cells were treated as in A and processed for EM. Cristae width (E) and the number of mitochondria with intramitochondrial vesicles (F) quantified as the average of 3 experiments ± S.D. ** p<0.01, * p<0.05. Representative images are shown in D. Asterisks denote intramitochondrial vesicles and arrowheads, sites where cristae are connected to both sides of a mitochondrion.

Mitochondria were marked using the outer membrane marker TOM20 and the matrix marker mtHSP70. In control cells, the two signals mostly overlapped (Fig 6A). In contrast, distinct distribution patterns could be observed in AA-treated cells independently of outer membrane fragmentation (Fig 6A), consistent with AA altering cristae structure [38]. As both *E*_*C*_ and *J/E* provide a very sensitive measure of changes in the mitochondrial network, we used the difference in these parameters between inner and outer membrane markers as a measure of changes in mitochondria inner structure. We first calculated the *E*_*C*_(TOM20)/*E*_*C*_(mtHSP70) ratio in control cells with overlapping signals for the two mitochondrial markers. In these cells, the *E*_*C*_ ratio was 1.1±0.2 (Fig 6B), close to the theoretical ratio of 1 for identical signals. Importantly, the ratio was significantly higher for AA-treated cells showing matrix to outer membrane changes (Fig 6B). A similar change was observed when the *J/E* ratio was used (Fig 6B), consistent with AA inducing changes in the inner membrane structure. Altogether, these results suggest that that this could provide a useful method to assess changes in the inner structure of mitochondria.

Since AA but not Myxo caused a significant shortening of the mitochondrial network, we used this method to determine whether Myxo nevertheless caused changes in cristae/matrix structure. As shown in Fig 6C, AA and Myxo caused a similar shift in the *J/E* ratio suggesting that, despite causing minimal fragmentation, Myxo promotes the rearrangement of the internal structure of mitochondria. We then used electron microscopy (EM) to validate the results obtained using confocal microscopy. Consistent with Complex III inhibition altering cristae structure, both treatments caused an increase in cristae width (Fig 6D and 6E). AA and Myxo also caused the appearance of vesicular structure within mitochondria (Fig 6D, asterisk; Fig 6F, quantification) that are consistent with the presence of septums/onions, structures separating the matrix into two discontinuous compartments [39]. These septums were also observed as cristae connected to both sides of a mitochondrion in AA and Myxo-treated cells, but not in control cells (Fig 6D, Arrowheads). Altogether, this data indicates that Complex III inhibition causes cristae rearrangements, supporting our results using confocal microscopy.

## Discussion

Modulation of mitochondrial length, connectivity and cristae structure plays a key role in important cellular processes such as metabolism, cellular differentiation and cell death [1,2,4,5]. One crucial aspect of mitochondrial function that has emerged in recent years is the tight correlation between mitochondrial length, cristae structure and mitochondrial bioenergetics [8,10,11,31]. In particular, amino acid starvation promotes both mitochondrial elongation and tightening of cristae structure, which sustain mitochondrial ATP production and prevent cell death [8,10,11]. However, as most existing algorithms were designed to measure apoptotic mitochondrial fragmentation [20,21,22], we still lack the proper tools to quantify stress-induced increases in length and connectivity.

Because elongated mitochondria overlap and connect to each other, the accurate identification of the actual topology of the network is highly challenging. To solve this issue, we used a probabilistic approach to measure both mitochondrial length distribution and connectivity. This approach has several advantages. First, it provides a global view of the mitochondrial network without having to rely on prior knowledge of the type of mitochondrial shapes present or what constitutes an elongated mitochondrion. This thus provide a much more objective representation of mitochondrial length than the manual binning relying on the estimation of the overall proportion of long and connected mitochondria within each cell. In addition, while some of the previously published automated methods give information on mitochondrial length, they only measure the distance between branch points [20,21,22,23,40], underestimating their actual length (see Fig 1B).

A second important feature of the algorithm is that it provides sensitive metrics to measure connectivity, an independent property of mitochondrial network altered in response to stress. For example, the mitochondrial fragmentation observed following mitochondrial impairment or apoptosis [30,41] is associated with a loss of mitochondrial connectivity caused by the accumulation of short isolated mitochondria (Blobs) (Fig 5). These contain only 3 structural elements (1 tubule, 2 ends) and lack junctions (*J/E* ratio equal to 0), leading to low *E*_*C*_ and *J/E* values. In contrast, stresses such as starvation increase both length and connectivity of mitochondria (Fig 4) [8, 12]. Interestingly, when length and connectivity are plotted for individual cells, distinct cells populations become apparent (Fig 5J). Specifically, while some cells show the hyperfused phenotype previously described (elongated and more connected [12]), others elongate their mitochondrial network without significant changes in their connectivity, or increase their connectivity without significantly changing their mitochondrial length (Fig 5J).

Two roles have been proposed for stress-induced fusion: facilitating the distribution of metabolite across the mitochondrial network and preventing mitophagy [8,11,29]. A fused mitochondrial network greatly improves the diffusion of both fatty acids and calcium throughout the cell, facilitating calcium signalling and improving bioenergetics [29,42]. As increased connectivity is likely to play a similar role, the main function of mitochondrial elongation could then be to prevent mitophagy, not improving bioenergetics. This is consistent with the observation that OPA1 dynamically regulates cristae structure independently of fusion to promote ATP synthase assembly and ATP production [10].

The distinction between elongation and connectivity is made possible by the use of sensitive measures of connectivity such as the new parameters we describe (*E*_*C*_, *J/E*). Previous work has relied on a number of descriptors of mitochondrial shape to measure changes in mitochondrial shape/connectivity [20,21,22]. Of these, aspect ratio and shape descriptors (blobs, donuts) do not measure connectivity [21], while other parameters such as connection/length or Formfactor/Roundness are not very sensitive measures of connectivity (Fig 3G) [21, 22]. In contrast, the parameters described here, namely *E*_*C*_ and *J/E*, changed dramatically with alterations in network topology in both the computer-generated images and actual cells. Given the sensitivity of these parameter, we used them to measure not only changes in mitochondrial connectivity occurring in response to stress, but also the resulting alterations in cristae/matrix. Such assessment of changes in cristae structure is currently time consuming, as it requires EM analysis. While EM remains the gold standard for cristae measurement, the algorithm could be used to rapidly screen for specific modulators of cristae morphology.

Another important aspect of mitochondrial dynamics is that the mitochondrial network changes over time in response to various intracellular and extracellular cues. In that context, while we performed the measurements of mitochondrial network (length, connectivity, internal structure) in fixed cells, the algorithm can just as easily measure changes occurring over time in the same cell to determine the kinetics of these changes. In fact, the only requirement for the use of the algorithm is to provide properly segmented images for the analysis. Numerous segmentation methods exist that are adapted to different type of images (see for example [16,19,21]). Here, we used a specific segmentation method that worked well in our experimental setting. Importantly, while the algorithm can be used irrespective of the segmentation method used, experimental validation of the segmentation algorithm is crucial to obtain an accurate measurement of mitochondrial networks.

In recent years, key proteins involved in the biogenesis of cristae and their maintenance have been identified (MICOS complex, OPA1) [43, 44, 45]. These can be modulated through metabolic signals such as changes in metabolic intermediates [43,44]. On the other hand, outer membrane fusion is achieved through the regulation of the fission protein DRP1, which is recruited to mitochondria during apoptosis but inactivated following starvation [8,11,41]. While changes in outer and inner membranes need to be coordinated to allow proper mitochondrial function, the underlying mechanisms remain poorly understood. Here we show that mechanistically distinct Complex III inhibitors, AA and Myxo [46], induce different changes in the mitochondrial network, only AA causing widespread mitochondrial fragmentation. Thus, our results indicate that loss of mitochondrial activity is not sufficient to cause mitochondrial fragmentation. In addition, while only AA caused mitochondrial fission, both inhibitors induced rearrangements in cristae/matrix structure, indicating that cristae rearrangement can occur independently of changes in outer membrane fusion. The presence of several distinct but overlapping mitochondrial responses (length, connectivity, cristae structure) in response to stress support the presence of a complex network of sensors and regulators of cristae structure that act in part independently of DRP1-driven outer membrane fission. Such a network would include the MICOS complex, OPA1, a key inner membrane fusion protein that is directly regulated by metabolite levels [10], as well as cytosolic sensors such as PKA and AMPK [8,30].

In conclusion, we have developed a novel algorithm based on a probabilistic approach to measure mitochondrial length and connectivity. This algorithm will provide a powerful tool to address the physiological relevance of mitochondrial dynamics in a wide range of settings.

## Methods

Cell culture reagents were obtained from Wisent. Other chemicals were purchased from Sigma-Aldrich, except where indicated.

### Cell culture, electron microscopy and immunofluorescence

WT Mouse Embryonic Fibroblasts (MEFs) and human U2OS cells were cultured in Dulbecco’s modified Eagle’s medium (DMEM) supplemented with 10% fetal bovine serum. For electron microscopy, cells were fixed in 8% glutaraldehyde in 0.4 M sodium cacodylate buffer as described [33], then shipped to Mount Sinai Hospital (Toronto, Canada) for processing. Images were acquired using an EMS 208S electron microscope (Philips). For immunofluorescence, cells were grown directly on glass coverslips and incubated for four hours in Earls Balanced Salt Solution (EBSS), Antimycin A (50 μM) or myxothiazol (1 μm) where indicated. Cells were then washed in PBS and fixed for 10 minutes in 4% paraformaldehyde. Mitochondria were stained with an antibody against TOM20 (FL-145, Santa Cruz Biotechnologies) or mtHSP70 (ABR Bioreagents) and AlexaFluor-conjugated secondary antibodies (Jackson Immunoresearch). Images were acquired with a Leica TSC SP8 confocal microscope fitted with a 63x/1.40 oil objective using the optimal resolution for the wavelength used (determined using the Leica software).

### Image segmentation

The algorithm requires as input a binary image representing the signal from the confocal microscope. This segmentation was done in ImageJ using the following procedure. Individual cells were first cropped from the original images to allow analysis on a cell-to-cell basis and to facilitate the analysis by the algorithm (smaller number of clusters/image). The initial confocal signal was first smoothed out using Median (2 pixels), then contrast was enhanced (0.4% saturated pixels; normalised, equalised histogram). A first binary image was then generated using the Auto Local Threshold plugin (Median 10; 10 pixels being the width of a mitochondrion). This image contains the area with mitochondrial information but also includes many areas of very low-intensity value. To overcome this problem, a second image was generated by manually setting a global threshold that includes all of the area with actual mitochondria. As this second thresholding method still includes some background pixels as signal, we then multiplied the two images, resulting in a final image where only the actual mitochondria information is preserved. Finally, any residual background pixels were removed by eroding this image (1 iteration, 4 counts). The method was robust across experimental conditions and not very sensitive to the global threshold value, as determined by manual evaluation of the images and the small variability of *J/E* with varying threshold values (S2 Fig).

### Synthetic images

The synthetic images consisted in 500 by 500 pixels images containing a given number of randomly generated mitochondria for which the scale was set so that 15 pixels = 1 μm, similar to the confocal images. Each image was defined by a length distribution and a total number of mitochondria, which are the two inputs used to generate the images. To create a mitochondrion, we randomly generated a point in the image independently of other already existing mitochondria. The mitochondrion was then elongated from this point using a variable direction to reproduce mitochondrial tortuosity. The process was repeated until we obtained the selected number of mitochondria within the defined range of lengths. A Gaussian intensity profile was added to the mitochondrial skeleton to increase mitochondrial width similarly to actual mitochondria. Each image was then segmented using a global threshold. Mitochondrial densities were measured from segmented images using ImageJ.

To compare the initial synthetic images to the skeleton generated by the algorithm, we verify two conditions: 1) that the elements of the skeleton are at the same place in both images and 2) that individual mitochondria have the same length. Since condition 1 is respected in >97% of the images, the observed variation is mostly due to changes in length. Assuming similar distributions (condition 1), the ratio is calculated by dividing the total length of the initial mitochondria in the image by the total length of the final skeleton generated by the algorithm.

### Algorithm

The algorithm (Momito) was coded in Java to be compatible with ImageJ and is freely available by contacting the authors or at www.uqtr.ca/LaboMarcGermain. The output for length data and parameters were saved as csv files. To define mitochondrial length and connectivity, the algorithm first generates a graph representing the topology of the mitochondrial network (Step 1, Fig 1C and S3 Fig). For this, a process of skeletonization (from ImageJ) is used to extract the centerline of mitochondria, thereby reducing the complexity of the image while keeping its topology. This image is used to assign a type of structural element (mitochondrial tubule, end or junction) to each pixel within the image. Adjacent pixels of the same type are then merged using an iterative process to generate the final map of structural elements. The structural elements are finally separated into clusters representing mitochondria connected together (Step 2, Fig 1C and S3 Fig). The connectivity parameters (*E*_*C*_, *J/E*) are compiled at this step. Of note, while three-way junctions mostly represent actual mitochondrial junctions, 4-way junctions are very likely the result of two independent mitochondria crossing each other [47]. 4-way junctions are thus disconnected by the algorithm before further analysis (S3 Fig, Step 1, Green element).

To determine the length profile of the mitochondrial network, the first step is to define its topology. As the exact pattern of connection between the different structural elements within a cluster cannot be directly defined, we determined the different possible connections within each cluster. This is done by sampling elements within a cluster and “walking” along the different elements to connect them, mapping the different possible paths that can be taken. This process generates the set of possible network configurations (Step 3, S3 Fig).

Specifically, each structural element has a defined set of possible topologies. For example, a structural element with three connections (a junction) has 17 considered interpretations that are given the same probability to simplify the analysis. The interpretation of a cluster is done by opening a tree in which each node represent a structural element that is connected to nodes in the subsequent level according to its number of possible connections. Given the large number of ways to connect nodes, the size of the tree increases exponentially with the number of elements in a cluster. To maximize the speed of the analysis, the algorithm randomly samples branches of the tree. The sampling can thus be seen as a tree traversal problem where we need to symmetrically open a large number of leaves of the tree while minimizing the number of leaves consisting of impossible interpretations. The configuration of each cluster is then calculated by averaging the possible interpretations. In addition, the length interpreter minimizes the impact of skeletonization on the length of interpreted mitochondria. This generates probability graphs for mitochondrial length and the number of mitochondria in the cluster (Step 3, Fig 1C and S3 Fig).

Finally, all the statistics for the image are combined (Step 4, Fig 1C and S1 Fig). To do this, the length distribution probability of each cluster is summed according to its weight in the overall image. This normalisation is done by considering the number of mitochondria in each cluster relative to the total number of mitochondria in the image (Distribution for the image = ∑(length profile/cluster x mitochondria/cluster)). The uncertainties for the ratio of each length are finally computed.

### EMD

EMD values were calculated using a previously described method [26,27,28]. Because EMD values are a relative distance between two distributions, the first step is to calculate the experimental variability, i.e. the differences in mitochondrial length distribution within each treatment condition across the various experiments (n). For an experimental design with two conditions (Control (C) and Treated (T) cells), we compare the distribution for one condition for each experiment to the average for that condition across experiments. For 3 experiments, we have C1, C2 and C3 that are individually compared to Average(C1, C2, C3), generating a EMD value for each comparison. EMD values are also calculated for T1, T2, T3 versus Average(T1, T2, T3). The change in distribution caused by the treatment is then measured by calculating EMD values for each condition against the average of the other. In the example, C1, C2 and C3 are compared to Average(T1, T2, T3) and T1, T2, T3 are compared to Average(C1, C2, C3). The statistical analysis is then done on the EMD values comparing the same condition (experimental variability) to the EMD values across conditions.

### Statistical analysis

For all experiments with cells except the sample images (Fig 3) and the individual ratios in Fig 6B, individual cells within an experiment (at least 20 cells/condition) were averaged before comparing these averages across experiments. This was done to take into account the experimental variability across experiments. Data is expressed as average ± SD. Variance between groups was similar in all cases. Normal distribution of the data was determined using a normal probability plot test. Statistical significance was determined using Student’s t test (between 2 groups) or one-way ANOVA with a tukey *post hoc* test (multiple comparisons).

## Supporting information

S1 Fig Skeletonization is similar across the range of mitochondrial densities found in cells. (A-B) Representative segmented cell showing areas of low (1; cell periphery) and high (2; preinuclear) mitochondrial densities. Inserts show the comparison between actual cells (Left) and synthetic images containing 100 or 500 mitochondria (Right). Mitochondrial densities are quantified in (B). L: mitochondrial density in the cell periphery; H: mitochondrial density in the perinuclear region; 100–500: mitochondrial densities in synthetic images with the indicated number of mitochondria. (C) Validation of the skeletonization process using synthetic images. Synthetic images containing increasing numbers of mitochondria (increasing mitochondrial area) were generated. The 1 pixel wide mitochondrial skeletons from the original images (pre-processing, pre) were then compared to the skeleton resulting from the processing of the images by the algorithm (post-processing, post). A pre/post ratio of 1 denotes mitochondria with the same length and position. Each point represents an individual image. The blue and red bars represent the average mitochondrial densities in the cell periphery and perinuclear region respectively (from (B)). (D) Total number of clusters analysed in the images form the different treatments used in our experiments. Data is expressed as the average (in percent of total clusters) of at least 3 experiments ± SD.
(TIF)

S2 Fig Validation of the segmentation process. (A) The image at the top was segmented using different global threshold values (bottom images), where “threshold” indicates the manually determined optimal threshold for that image and the other values, the variation from this optimal threshold. (B) The effect of threshold variation on the algorithm output was determined by measuring the *J/E* value of each image (n = 5/condition) in relation to changes in the threshold value used. To allow comparison between images, all *J/E* values were normalised to the *J/E* value at the optimal threshold.
(TIF)

S3 Fig Details of the analysis process. The numbered steps on the left side correspond to the steps in Fig 1C. SE, Structural Element; C1-C3, Clusters 1–3; I_1_-I_n_, Interpretation 1-n. The green structural element in the SE box represents a 4-way junction that is disconnected by the algorithm, as this type of connection most likely represent two overlapping mitochondria rather than a junction. See Methods for details.
(TIF)
