# Supplementary figures and images for: A novel algorithm identifies stress-induced alterations in mitochondrial connectivity and inner membrane structure from confocal images
Source: PLoS Comput Biol. 2017 Jun 22;13(6):e1005612. doi: 10.1371/journal.pcbi.1005612 (PMC5501662; doi:10.1371/journal.pcbi.1005612)

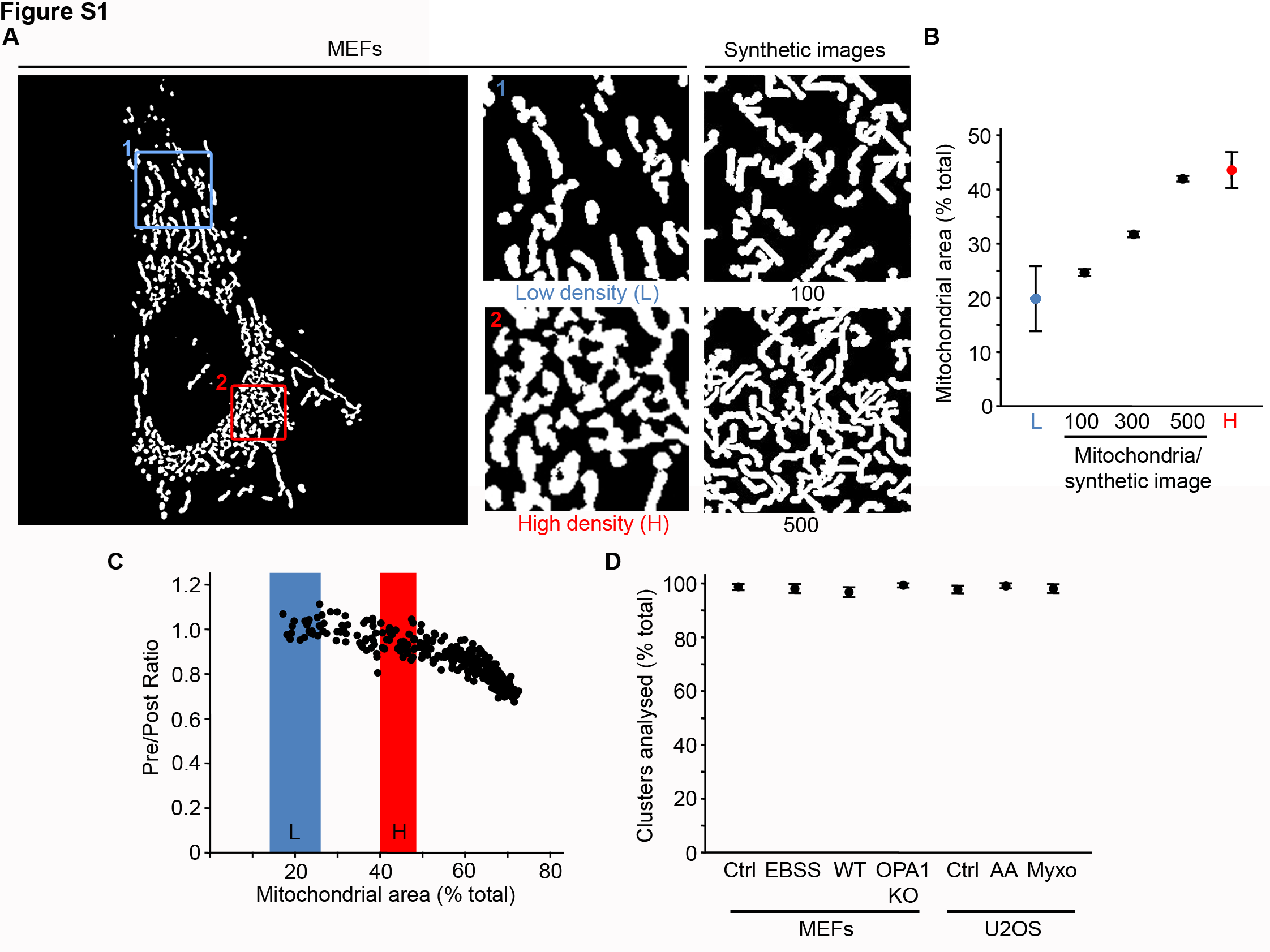

Supplement: S1 Fig — (A-B) Representative segmented cell showing areas of low (1; cell periphery) and high (2; preinuclear) mitochondrial densities. Inserts show the comparison between actual cells (Left) and synthetic images containing 100 or 500 mitochondria (Right). Mitochondrial densities are quantified in (B). L: mitochondrial density in the cell periphery; H: mitochondrial density in the perinuclear region; 100–500: mitochondrial densities in synthetic images with the indicated number of mitochondria. (C) Validation of the skeletonization process using synthetic images. Synthetic images containing increasing numbers of mitochondria (increasing mitochondrial area) were generated. The 1 pixel wide mitochondrial skeletons from the original images (pre-processing, pre) were then compared to the skeleton resulting from the processing of the images by the algorithm (post-processing, post). A pre/post ratio of 1 denotes mitochondria with the same length and position. Each point represents an individual image. The blue and red bars represent the average mitochondrial densities in the cell periphery and perinuclear region respectively (from (B)). (D) Total number of clusters analysed in the images form the different treatments used in our experiments. Data is expressed as the average (in percent of total clusters) of at least 3 experiments ± SD. (TIF) [file pcbi.1005612.s001.tif]

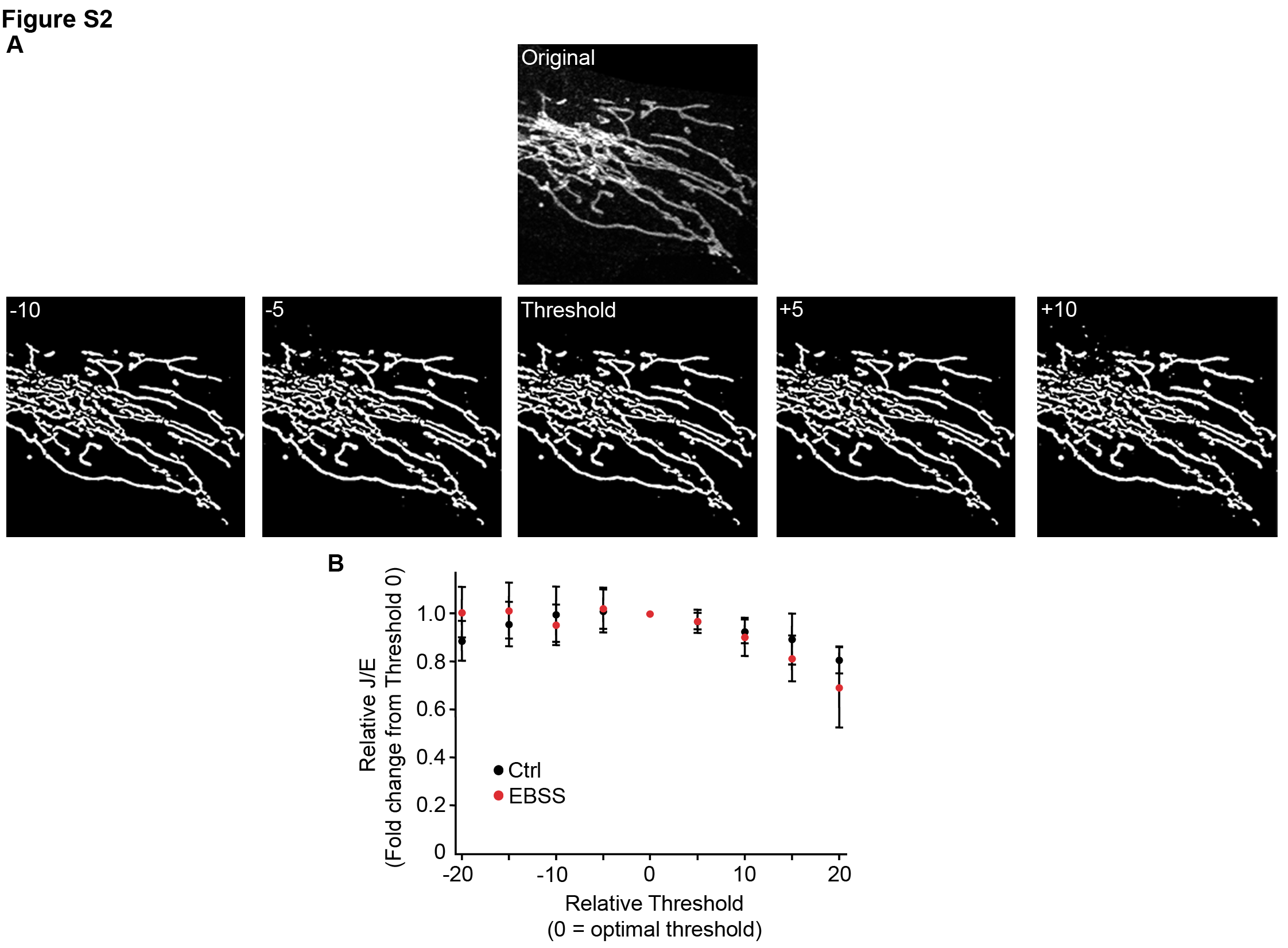

Supplement: S2 Fig — (A) The image at the top was segmented using different global threshold values (bottom images), where “threshold” indicates the manually determined optimal threshold for that image and the other values, the variation from this optimal threshold. (B) The effect of threshold variation on the algorithm output was determined by measuring the J/E value of each image (n = 5/condition) in relation to changes in the threshold value used. To allow comparison between images, all J/E values were normalised to the J/E value at the optimal threshold. (TIF) [file pcbi.1005612.s002.tif]

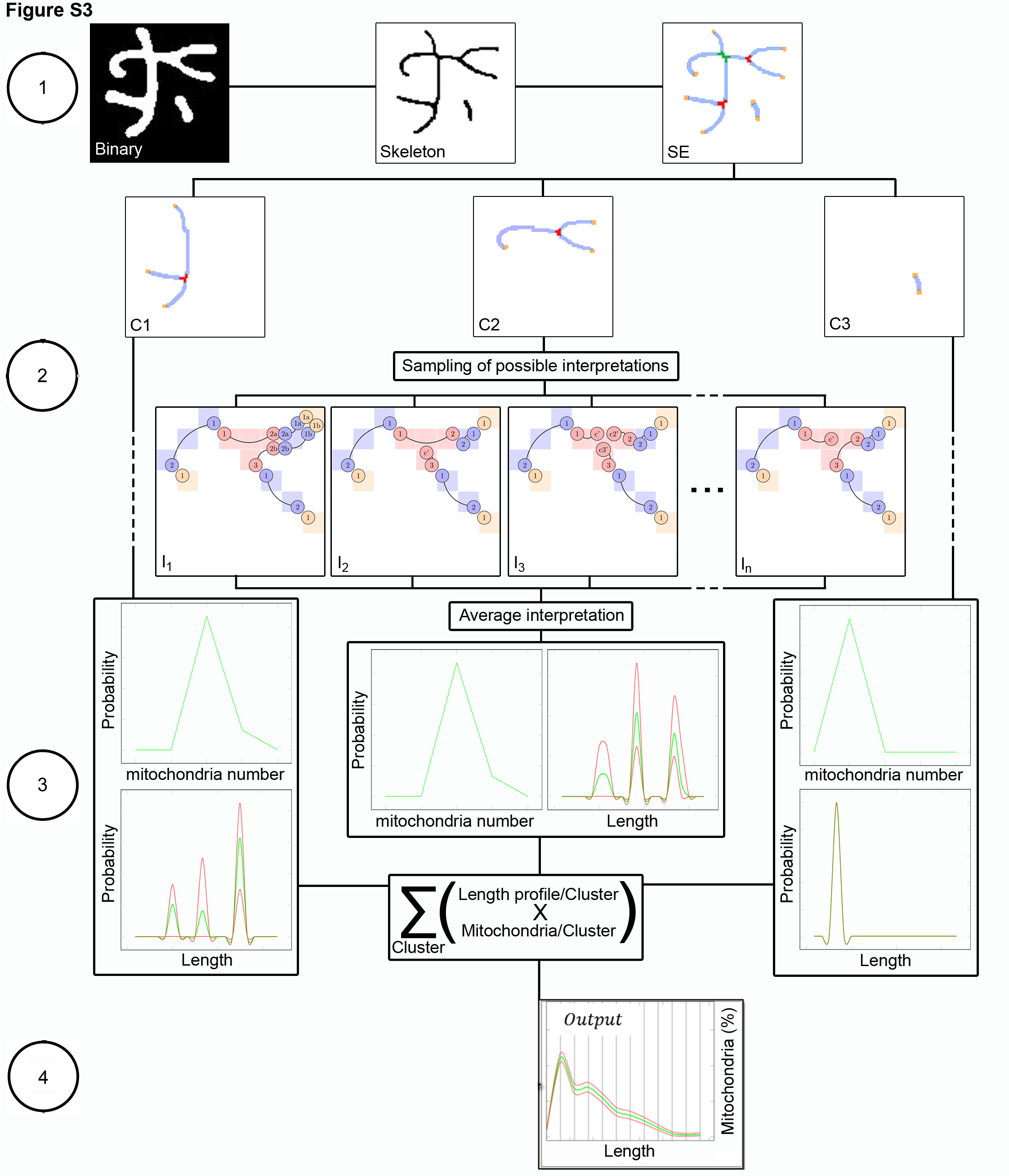

Supplement: S3 Fig — The numbered steps on the left side correspond to the steps in Fig 1C. SE, Structural Element; C1-C3, Clusters 1–3; I1-In, Interpretation 1-n. The green structural element in the SE box represents a 4-way junction that is disconnected by the algorithm, as this type of connection most likely represent two overlapping mitochondria rather than a junction. See Methods for details. (TIF) [file pcbi.1005612.s003.tif]
